# TRPM4 Participates in Aldosterone-Salt-Induced Electrical Atrial Remodeling in Mice

**DOI:** 10.3390/cells10030636

**Published:** 2021-03-12

**Authors:** Christophe Simard, Virginie Ferchaud, Laurent Sallé, Paul Milliez, Alain Manrique, Joachim Alexandre, Romain Guinamard

**Affiliations:** EA 4650, Signalisation, Electrophysiologie et Imagerie des Lésions d’Ischémie-Reperfusion Myocardique, GIP Cyceron, Université de Caen Normandie, CHU de Caen, 14032 Caen, France; christophe.simard@unicaen.fr (C.S.); ferchaud-v@chu-caen.fr (V.F.); laurent.salle@unicaen.fr (L.S.); milliez-p@chu-caen.fr (P.M.); manrique@cyceron.fr (A.M.); joachim.alexandre@unicaen.fr (J.A.)

**Keywords:** TRPM4, aldosterone, atria, atrial arrhythmias

## Abstract

Aldosterone plays a major role in atrial structural and electrical remodeling, in particular through Ca^2+^-transient perturbations and shortening of the action potential. The Ca^2+^-activated non-selective cation channel Transient Receptor Potential Melastatin 4 (TRPM4) participates in atrial action potential. The aim of our study was to elucidate the interactions between aldosterone and TRPM4 in atrial remodeling and arrhythmias susceptibility. Hyperaldosteronemia, combined with a high salt diet, was induced in mice by subcutaneously implanted osmotic pumps during 4 weeks, delivering aldosterone or physiological serum for control animals. The experiments were conducted in wild type animals (*Trpm4^+/+^*) as well as *Trpm4* knock-out animals (*Trpm4^-/-^*). The atrial diameter measured by echocardiography was higher in *Trpm4^-/-^* compared to *Trpm4^+/+^* animals, and hyperaldosteronemia-salt produced a dilatation in both groups. Action potentials duration and triggered arrhythmias were measured using intracellular microelectrodes on the isolated left atrium. Hyperaldosteronemia-salt prolong action potential in *Trpm4^-/-^* mice but had no effect on *Trpm4^+/+^* mice. In the control group (no aldosterone-salt treatment), no triggered arrythmias were recorded in *Trpm4^+/+^* mice, but a high level was detected in *Trpm4^-/-^* mice. Hyperaldosteronemia-salt enhanced the occurrence of arrhythmias (early as well as delayed-afterdepolarization) in *Trpm4^+/+^* mice but decreased it in *Trpm4^-/-^* animals. Atrial connexin43 immunolabelling indicated their disorganization at the intercalated disks and a redistribution at the lateral side induced by hyperaldosteronemia-salt but also by *Trpm4* disruption. In addition, hyperaldosteronemia-salt produced pronounced atrial endothelial thickening in both groups. Altogether, our results indicated that hyperaldosteronemia-salt and TRPM4 participate in atrial electrical and structural remodeling. It appears that TRPM4 is involved in aldosterone-induced atrial action potential shortening. In addition, TRPM4 may promote aldosterone-induced atrial arrhythmias, however, the underlying mechanisms remain to be explored.

## 1. Introduction

Hyperaldosteronemia was shown to be associated with atrial fibrillation in humans [1], which was reproduced in rats [2]. This may occur through different pathways since atrial fibrillation is known to have multifactorial determinants [3]. It includes structural abnormalities such as fibrosis; modifications of cell electrical properties with impaired action potential duration due to modification of ion channels expression or regulation; rearrangement of cell-to-cell communication after a modification of connexins expression; and dysregulation of Ca^+^-handling. Interestingly, patients with atrial fibrillation also exhibit an increase in the mineralocorticoid receptor (MR) expression in atrial cardiomyocytes, which may enhance the effect of aldosterone [4]. MR activation was shown to increase the expression of a variety of ion channels in cardiomyocytes, particularly in those participating in action potential such as the Na^+^, K^+^, and Ca^2+^ voltage gated channels [4,5,6,7]. On the other hand, MR activation also increases Ryanodine receptor activation without altering its expression [8]. In combination with the increase in Ca^+^ influx, it contributes to a perturbation of Ca^+^-transient and thus arrhythmias. Finally, aldosterone at high levels as well as MR activation also decrease connexin-43 (Cx43) expression and assembly into gap-junction plaques which, once again, may contribute to arrhythmias [9,10].

Ion channels expression profiles are different between atrial and ventricular cardiomyocytes. The Transient Receptor Potential Melastatin 4 (TRPM4) channel which is expressed in mice, rats, as well as human cardiomyocytes [11,12,13,14] exhibits a higher expression at the atrial level compared to the ventricular level. In mice, TRPM4 was shown to participate in atrial action potential (AP) duration since its pharmacological inhibition shortened AP, which was observed neither in transgenic *Trpm4* knock-out animals (*Trpm4^-/-^*) nor in ventricular cardiomyocytes [15]. TRPM4 belongs to the TRP family with a variety of members expressed in cardiac tissue [16]. It is a non-selective monovalent cation channel (equal permeability for Na^+^ and K^+^) that is widely expressed among mammalian tissues with a high expression in the heart [13,17,18]. Its main regulation is a sensitivity to internal Ca^+^ [17,19]. According to this, it is known to participate in Ca^+^ handling first since it modulates the driving force for Ca^+^ entry by producing a depolarizing current and secondly because it is itself modulated by internal Ca^+^. In addition, TRPM4 was shown to be implicated in hypoxia and reoxygenation arrhythmias such as early-after depolarization (EAD) in mouse, a condition known to be associated with Ca^+^-transient modifications [20].

Altogether it indicates that, on the one hand, hyperaldosteronemia induces atrial remodeling at the morphological and electrical level with Ca^+^ transient and AP duration impairment. On the other hand, TRPM4 is known to influence and be influenced by Ca^+^ transient and participate in atrial AP duration. It also influences cardiac morphological remodeling. We thus hypothesized that the effect of aldosterone could involve TRPM4 for morphological modifications as well as electrical perturbations leading to atrial arrythmias.

The aim of our study was to elucidate the potential interaction between the aldosterone pathway and TRMP4 in left atrial (LA) structural remodeling, AP modulation, and arrhythmias. 

## 2. Methods

### 2.1. Ethical Approval

Experiments were carried out in strict accordance with the European Commission Directive 2010/63/EU for animal care. They were conducted with authorization for animal experimentation by the local committee (Comité d’Éthique Normandie en Matière d’Expérimentation Animale, CENOMEXA, registration # C2EA-54, referral # 13-257).

### 2.2. Animal Model

Knockout mice (*Trpm4* ^-/-^) and littermate controls (*Trpm4* ^+/+^) from a C57BL/6J strain were obtained as described previously [20]. Experiments were conducted on 2-month-old males. The genotype was confirmed by genomic PCR performed on tail DNA with primers specific for the wild-type and null alleles according to previously reported proceedings [21].

Mice were housed in cages to European standards (type IV), with a pathogenic free and controlled environment (21 ± 1 °C; humidity 60%; pressure 20–25 Pa; lights on 6:45 AM to 6:45 PM; enriched environment). Food and water were available ad libitum and were changed once a week, as was the litter with 5 mice per cage. All efforts were made to minimize animal suffering and number.

Hyperaldosteronemia and a high sodium diet was induced in the AL+S groups according to the model previously described [22]. It combines the continuous administration of aldosterone by implanted osmotic minipumps with enhanced dietary NaCl (drinking water supplemented with 1% NaCl) and left nephrectomy. Minipumps (ALZET^®^, Laboratoire Charles River^®^, Lyon, France) were implanted subcutaneously on the back of 8-week animals under anesthesia with isoflurane. The minipumps were previously filled with 0.1 mg of aldosterone (Sigma-Aldrich, L’isle d’Abeau, France) diluted in a sterile physiological serum supplemented with 5% ethanol (total volume 0.2 mL) or with only physiological serum and ethanol for the control group (CTRL). Note that the minipumps were preincubated for 24 h in a physiological serum before surgery to ensure the immediate efficiency after implantation. The minipumps output was 250 nLh^−1^. At the same time as the minipump implantation, the left nephrectomy was performed on all animals (CTRL and AL+S) after renal pedicle ligation. A high salt diet was used since it is known to increase aldosterone target-organ damages [22,23].

### 2.3. Echocardiography

Echocardiography was performed using an IE33 ultrasound system connected with a linear high frequency L15-7io transducer (Philips Healthcare, Best, the Netherlands). Two-dimensional images were used for classical echocardiographic measurements (see Table 1) and M-mode was used for the determination of the left atrial diameter in the parasternal long axis view. Measurements were performed in anesthetized animals (isoflurane 1.5% in 70% N_2_O + 30% O_2_) before minipump implantation (D0, 8-week-old animals) and after 28 days of implantation (D28). Animals were placed in a supine position, shaved using a chemical hair remover, and ultrasound gel was applied to optimize the detection of cardiac chambers. Heart rate was obtained from M-mode recordings.

### 2.4. Electrophysiology

Intracellular microelectrodes were used to record transmembrane potential of cardiomyocytes from left atrial mouse appendages after 28 days of incubation with physiological serum (CTRL) or aldosterone (AL+S), as indicated above.

Animals were euthanatized by cervical dislocation and the heart was quickly removed. The atria were then separated from the ventricles. Atria were pinned in a 5-mL superfusion chamber without being opened. The preparation was continuously superfused using a peristaltic pump with a physiological solution at the rate of 16 mL·min^−1^ bubbled with 95% O_2_ and 5% CO_2,_ and maintained at 37 °C. The physiological solution contained (in mmol·L^−1^): NaCl 108.2, KCl 4, CaCl_2_ 1.8, MgCl_2_ 1, NaH_2_PO_4_ 1.8, NaHCO_3_ 25, and glucose 11 (pH 7.35). The preparation was stimulated at a rate of 5 Hz by square electric pulses using an S88X Stimulator (GRASS, Astro-Med, RI, USA) connected to a bipolar electrode made with silver wires. All chemicals for microelectrodes experiments were from Sigma-Aldrich.

Action potentials (APs) were recorded after cell impalement using a glass microelectrode filled with KCl 3 mol·L^−1^ and with a tip resistance of about 10 megaohms. Microelectrodes were coupled to the input stages of a home-built impedance capacitance-neutralizing amplifier and connected to a PowerLab 4/26 A/D converter (ADinstruments, Paris, France). Recordings were displayed and analyzed using cardiac AP automatic acquisition software LabChart 7 (ADinstruments, Paris, France) providing the resting membrane potential (RMP), the action potential amplitude (APA), the action potential duration at 50% (APD_50_), 70% (APD_70_), and 90% (APD_90_) of repolarization, and the maximum upstroke velocity of AP during the depolarizing phase (V_max_). While the preparation was stimulated at 5 Hz, triggered arrhythmias (early after depolarizations (EAD) and delayed after depolarizations (DAD)) appeared in some experiments. The number of EADs and DADs was determined by the visual screening of the recordings. An event was considered as an EAD or DAD when additional depolarization occurred during AP repolarization, before completion of total repolarization or after repolarization, independently to the stimulation. The number of triggered arrhythmias was determined over 15 min periods. In another series of experiments, called stimulated arrythmias, EAD and DAD were induced by rapid pacing as described previously [24]. The preparation was stimulated for 10 s at 20 Hz. The number of EAD and DAD was determined during the following 5 min. The protocol was reproduced 3 times on each atria.

### 2.5. Plasmatic Aldosterone Measurement

On the day of the cardiac sample (D28), animals were anesthetized by intraperitoneal injection of 0.1 mL of Pentobarbital (TVM, Euthasol Vet, 400 g·L^−1^) and an intracardiac blood sample was collected. The blood sample was stored at 4 °C in EDTA then centrifugated at 3500 rpm for 15 min. The plasma sample was stored at −80 °C until analysis. Plasma aldosterone level was determined by liquid chromatography coupled with mass spectrometry using a QTRAP 50500 LC-MS/MS system (SCIEX, Villebon sur Yvette, France).

### 2.6. Immunolabeling

As mentioned for the plasma aldosterone sample, animals were anesthetized with a pentobarbital injection after 28 days of minipump treatment. A thoracotomy was performed to allow heart dissection. The left atrium was isolated and placed in 4% formalin then fixed in paraffin. Six μm slices were cut using a microtome and placed on microscope slides. Nucleus were labeled using hematoxylin. Cx43 immunolabeling was done with rabbit polyclonal antibody NeoBiotech NB22-2409.

The number of organized Cx43 clusters were done by visual analysis by counting of the number of clusters in an image of 0.06 mm^2^. A total of 10 different images were counted for a single atrium from at least 2 slices. Data were reported as number of clusters by 0.1 mm^2^. We differentiated clusters at the junctional plaques which appeared as short continued lines of labeling with an orientation consistent with the junctional plaques and clusters at the lateral membranes which appeared as long dotted lines of immunolabeling.

Cell density was determined through visual analysis and by counting the number of nucleus in an image of 0.06 mm^2^. A total of 10 different images were counted for a single atrium from at least 2 slices. Data were reported as the number of cells by 0.1 mm^2^.

Endothelium thickness at the inside of the LA was evaluated by screening each slice to select an area with two apparent internal side of the atrium, thus providing two measurements for each slice. A total of 2 slices were used for a single atrium.

### 2.7. Fibrosis Staining

Fibrosis was evaluated using picro Sirius red (0.1% Sirius, 1.3% picric acid) (Raldiagnostics, Bordeaux, France) staining on 6-µm left atrial slices as described in the previous section. Four slices were analyzed for each atria. To measure staining intensity, tissue segmentation was manually performed using Aperio ImageScope software v12.3 (Leica Biosystem, Wetzlar, Germany). Data were expressed as the relative proportion of stained tissue to total tissue area.

### 2.8. Data Analysis

Results are reported as mean ± S.E.M. Normal distribution was tested using a Shapiro–Wilk test. For those passing the test a parametric t-test was thus applied to compare data from a different series of experiments. For those that did not have normal distribution, a non-parametric Wilcoxon–Mann Whitney test was used to compare data from a different series of experiments. To compare the number of atria with arrhythmias, a Fischer exact test was used. Statistically significant difference was achieved for values of *p* < 0.05. Statistically significant differences are indicated in the graphs by asterisks. N is used to refer to the number of mice used. For experiments in the left atrial slices, n refers to the number of slice sections analyzed and N refers to the number of animals.

## 3. Results

At the end of the protocol, the aldosterone level in *Trpm4^+/+^* mice was 0.36 ± 0.05 × 10^−9^ mol·L^−1^ in CTRL (left-nephrectomy) animals (*N* = 6) but rose to 5.88 ± 2.24 × 10^−9^ mol·L^−1^ in AL+S (left nephrectomy + aldosterone + NaCl) mice (*N* = 3). Similar results were obtained for *Trpm4^-/-^* animals (0.36 and 4.25 × 10^−9^ mol·L^−1^ in CTRL and AL+S, respectively, *N* = 2 for each group).

### 3.1. Left Atrial Structural Remodeling

**Figure 1:**
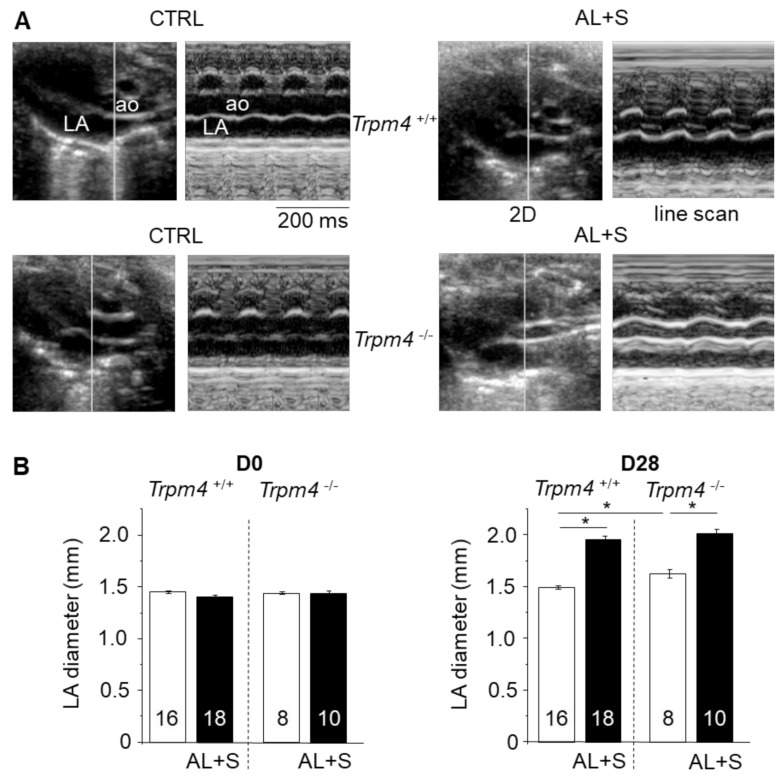
Left atrial structural remodeling after aldosterone + salt treatment. (**A**) Example of transthoracic echocardiography for *Trpm4^+/+^* and *Trpm4^-/-^* without (CTRL) or with (AL+S) 28 days aldosterone treatment on anesthetized 3-month-old female mice. The CTRL group corresponds to the control group with only the left nephrectomy. The AL + S group corresponds to the group with the left nephrectomy + aldosterone treatment and rich NaCl diet. For each group, the left panel indicates a representative B-mode image showing aorta (ao) and left atrium (LA) while the right panel shows the corresponding M-mode (cursor line indicated in the left panel). (**B**) Mean left atrial diameter according to the echography measurements before (D0) and after 28 days (D28) of treatment without (white) or with (black) aldosterone. Aldosterone produces a significant dilatation after 28 days. The numbers in the bars correspond to the number of animals. Picro Sirius red staining was used to search for fibrosis in left atrium slices at D28 for at least eight animals per group. Statistical significance between groups are indicated by *.

Echocardiography demonstrated a significant LA dilation after 28 days of infusion with aldosterone in both *Trpm4^+/+^* and *Trpm4^-/-^* mice when compared to the corresponding control group (Figure 1, Table 1). Note that a slight but significant LA dilation was detected in 3-month-old *Trpm4^-/-^-*CTRL mice compared to the *Trpm4^+/+^-*CTRL animals (*N* = 16 for +/+ and 8 for -/-), indicating an effect of *Trpm4* disruption on atrial development.

Atrial weight was also measured in harvested hearts after the completion of the protocol (28 days after minipump implantation). *Trpm4^+/+^-*CTRL and *Trpm4^-/-^-*CTRL mice had similar atrial weight (left and right atria) of respectively 7.2 ± 0.7 mg (*N* = 7) and 6.4 ± 0.4 mg (*N* = 9) which was increased by 17% (*N* = 5) and 27% (*N* = 7) in the AL+S groups without reaching significance (Table 2). Aldosterone induced a significant 8% increase in *Trpm4^+/+^* body weight which was not reproduced in *Trpm4^-/-^* animals but the tibia length was similar in all groups. Additionally, while right kidneys had a similar weight in *Trpm4^+/+^*-CTRL and *Trpm4^-/-^*-CTRL animals, and they significantly increased by 39% and 44%, respectively, in the AL+S groups (Table 2). No variation of fibrosis staining was observed due to 28 days aldosterone-salt treatment and/or *Trpm4* disruption (Supplementary data, Figure S1). 

### 3.2. Heart Rate

Under isoflurane anesthesia, *Trpm4^+/+^* and *Trpm4^-/-^*-CTRL animals exhibited a similar heart rate (453 ± 11 bpm (*n* = 16) and 455 ± 16 bpm (*n* = 7), respectively), in baseline conditions. However, while treatment with aldosterone + salt had no effect on the *Trpm4^+/+^* animals’ heart rate, it produced a significant 17% decrease in *Trpm4^-/-^* mice (Table 1).

### 3.3. Action Potential Parameters

**Figure 2:**
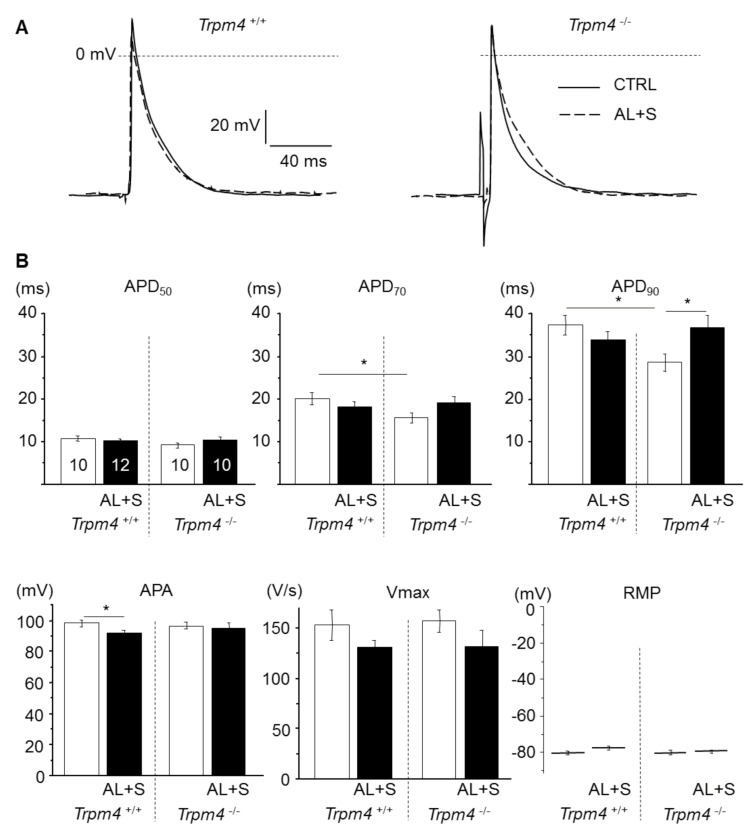
Effect of aldosterone + salt treatment on atrial action potential. (**A**) Representative action potentials (AP) from isolated left atrium recorded using intracellular microelectrodes for *Trpm4^+/+^* (left) and *Trpm4^-/-^* (right) animals, stimulated at 5 Hz. For each group, action potential from an aldosterone treated animal (dotted trace) is overlaid to a control animal (solid trace). (**B**) Mean AP parameters for each group (numbers of animals are indicated in the APD_50_ graph and are similar for other graphs). APD_50, 70, 90_ (action potential duration at 50, 70, and 90% repolarization); APA (action potential amplitude); V_max_ (upstroke maximal velocity); and RMP (resting membrane potential). Statistical significance between groups are indicated by *.

The effect of aldosterone treatment on LA action potential was investigated using intracellular microelectrodes. Under baseline conditions, similar cells RMP were measured for all groups (Figure 2). Representative APs and AP parameters are shown for all groups in Figure 2. For *Trpm4^+/+^-*CTRL animals, the APD_50_, APD_70_, and APD_90_, the APA and V_max_ were similar to those previously reported for mouse atrial AP [12,20]. *Trpm4^-/-^* mice exhibited a significantly shortened AP compared to *Trpm4^+/+^* animals, since APD_90_ was 28.6 ± 2 ms (*n* = 10) in *Trpm4^-/-^* but 37.4 ± 2.2 ms (*n* = 10) in *Trpm4^+/+^* while APA, V_max_, and RMP were similar to *Trpm4^+/+^* animals. The treatment by aldosterone and salt had no effect on AP duration from *Trpm4^+/+^* animals but prolonged AP in *Trpm4^-/-^* mice. It also induced a tiny reduction in APA in *Trpm4^+/+^* animals but not in *Trpm4^-/-^* (Figure 2).

### 3.4. Detection of Arrhythmias on Isolated Left Atrium

**Figure 3:**
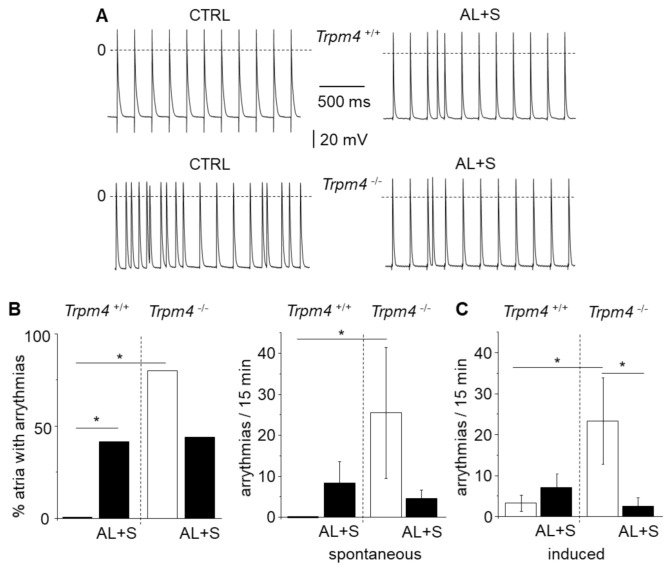
Occurrence of triggered arrhythmias on isolated left atria. (**A**) Representative recordings with action potentials from isolated left atrium recorded using intracellular microelectrodes for *Trpm4^+/+^* and *Trpm4^-/-^* animals stimulated at 5 Hz. Triggered arrhythmias (early after depolarization and delayed after depolarization) were recorded in both groups after aldosterone + salt treatment but only on *Trpm4^-/-^* animals without aldosterone + salt. (**B**) Percent of isolated left atria with arrhythmias (left) for both groups (*N* = 9 to 12 as indicated in the text) and mean number of arrhythmias (right) during 15 min. (**C**) Mean number of arrhythmias induced by rapid pacing (10 s at 20 Hz). Statistical significance between groups are indicated by *.

Triggered arrhythmias in the form of EAD and DAD were searched for during periods of 15 min. None of the atria from *Trpm4^+/+^-*CTRL mice exhibited arrhythmias (*N* = 11). However, 42% (*N* = 12) of the atria from *Trpm4^+/+^-*AL+S animals exhibited EAD or DAD (Figure 3). In experiments on *Trpm4^-/-^* mice, arrhythmias were detected even in the absence of aldosterone treatment, in 80% of atria (*N* = 10) and in 44% (*N* = 9) after aldosterone treatment which is not significantly different. When evaluating the number of arrhythmias for periods of 15 min, it appeared a significantly higher number of arrhythmias in *Trpm4^-/-^-*CTRL mice compared to *Trpm4^+/+^-*CTRL. The number of arrhythmias was reduced by aldosterone + salt treatment in *Trpm4^-/-^* animals while not reaching significance.

Atria were also stressed by rapid pacing (10 s at 20 Hz) to induce arrythmias. In these conditions 45% (*N* = 11) of atria from *Trpm4^+/+^-*CTRL mice and 58% (*N* = 12) of atria from *Trpm4^+/+^-*AL+S exhibited arrhythmias (N = 11). On *Trpm4^-/-^* mice, arrhythmias were detected in 78% (*N* = 9) of atria from *Trpm4^-/-^-*CTRL but 33% (*N* = 9) after aldosterone + salt treatment. The protocol was reproduced three times for each atria and arrythmias (EAD + DAD) were measured for 5 min after each stimulation. Data presented Figure 3C reports the total number of recorded arrythmias indicating a significant smaller occurrence of arrythmias in the *Trpm4^-/-^-*AL+S compared to the *Trpm4^-/-^-*CTRL group.

### 3.5. Connexin 43 Distribution

**Figure 4:**
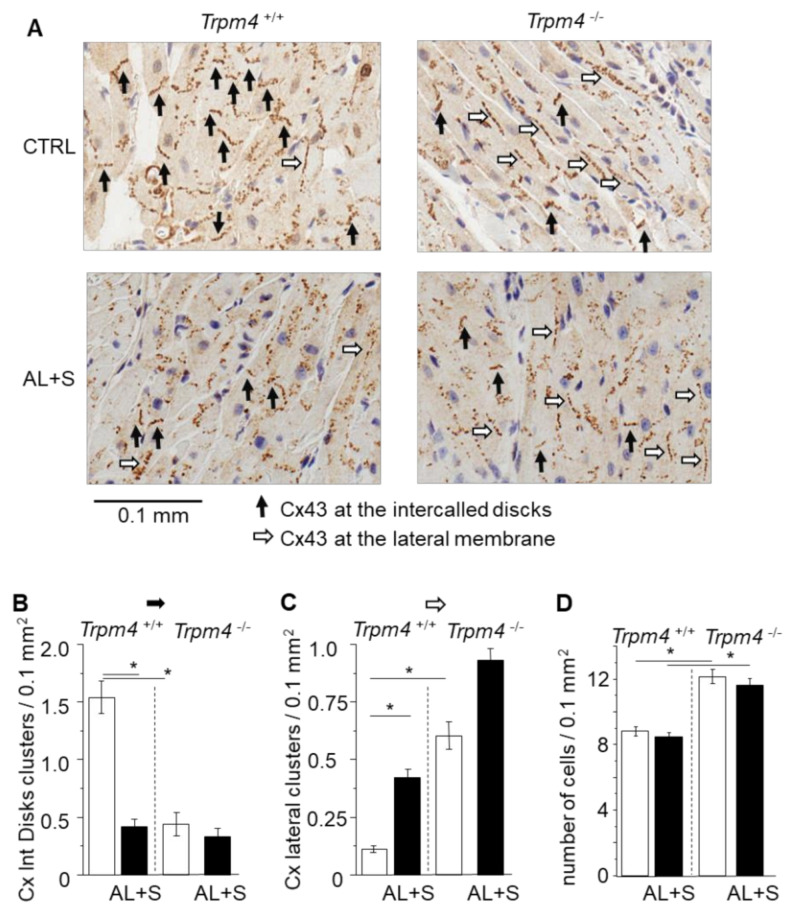
Organization of Connexin 43. (**A**) Representative immunolabelling of Cx43 (brown) in paraffin fixed embedded left atria sections (6 µm thickness) from *Trpm4^+/+^* and *Trpm4^-/-^* animals after 28 days of treatment with or without aldosterone + salt. Hematoxylin labelling (nucleus in blue). Black arrows indicate organized Cx43 complexes at the junctional plaques between two adjacent cardiomyocytes. White arrows indicate organized Cx43 complexes at the lateral membrane of cardiomyocytes. (**B**) Mean density of organized Cx43 complexes at the intercalated disks (organized Cx/0.1 mm^2^) (*n* = 20 to 50; *N* = 2 to 6 as indicated in the text). (**C**) Mean density of organized Cx43 complexes at the lateral membranes (organized Cx/0.1 mm^2^) (*n* = 20 to 50; *N* = 2 to 6 as indicated in the text). Aldosterone treatment as well as *Trpm4^-/-^* disruption induce Cx43 disorganization. (**D**) Mean cell density for each group (*n* = 20 to 50; *N* = 2 to 6). Statistical significance between groups are indicated by *.

Cx43 distribution was evaluated by measuring the number of well-organized clusters of Cx43 at the intercalated disks or at the lateral membrane, by surface area for each atrium (Figure 4). As pointed by black arrows in the examples provided in Figure 4, the clusters at the intercalated disks appeared as short continued lines of immunolabelling (black arrows) with an orientation consistent with the junctional plaques while clusters at the lateral membrane of cardiomyocytes appeared as long dotted lines of immunolabelling (white arrows). While *Trpm4^+/+^*-CTRL animals exhibited 1.5 ± 0.1 clusters by 0.1 mm^2^ at the intercalated disks (*n* = 50; *N* = 6), the number was significantly reduced by 73% (*n* = 40; N = 4) in the *Trpm4^+/+^*-AL+S animals. Inversely, the number of Cx43 clusters at the lateral membranes was increased by 2.7 folds in *Trpm4^+/+^-*AL+S compared to control animals. It indicated that aldosterone induced a Cx43 reorganization. *Trpm4^-/-^*-CTRL mice also exhibited a significantly lower number of organized cluster at the intercalated disks of 0.4 ± 0.1 by 0.1 mm^2^ (*n* = 20; *N* = 2), compared to *Trpm4^+/+^-*CTRL animals. A similarly low level was detected in *Trpm4^-/-^*-AL+S mice (*n* = 30; *N* = 3). Once again, this was accompanied by an increase of clusters at the lateral membranes (Figure 4).

### 3.6. Cell Number

Cell number by surface area was evaluated after nucleus labeling with hematoxylin on LA slices (Figure 4). *Trpm4^+/+^-*CTRL animals had a density of 8.8 ± 0.3 cells by 0.1 mm^2^ (*n* = 50; *N* = 6) which was not affected by aldosterone + salt treatment (*n* = 40, *N* = 4). *Trpm4^-/-^*-CTRL animals had a significant 37% higher cell density (*n* = 20; *N* = 2) compare to *Trpm4^+/+^-*CTRL. Cell density was not affected by aldosterone + salt treatment in *Trpm4^-/-^* (*n* = 30; *N* = 3) (Figure 4).

### 3.7. Endothelial Enlargement

**Figure 5:**
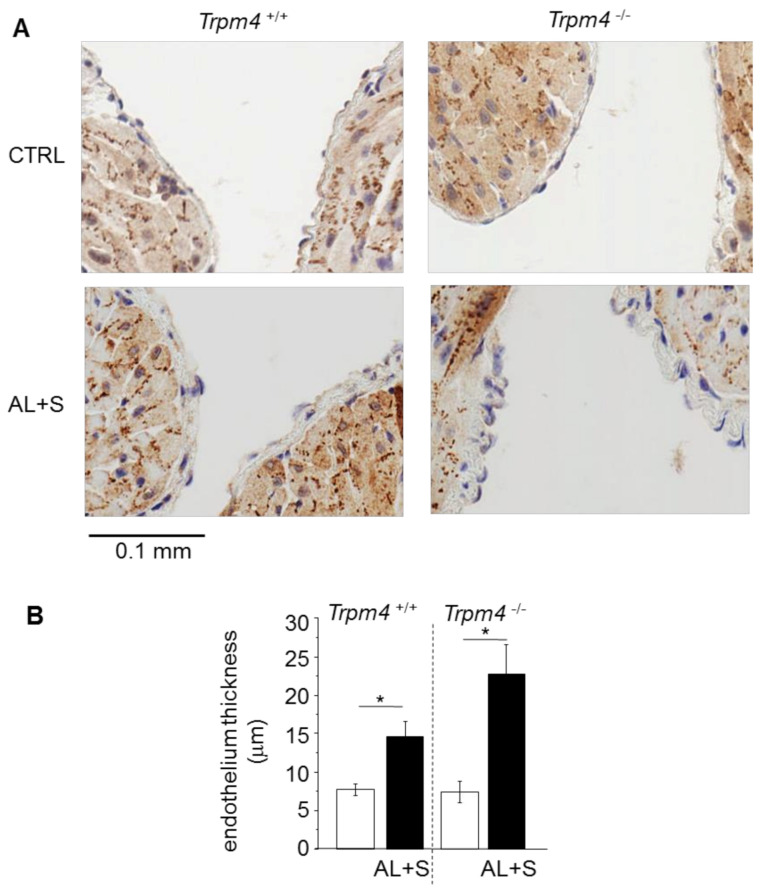
Endothelial enlargement. (**A**) Representative sections (6 µm thickness) of the left atrium showing the endothelium from Trpm4^+/+^ and Trpm4^-/-^ animals after 28 days of treatment with or without aldosterone + salt. Hematoxylin labeling (nucleus in blue). Pictures are selected to show the inside of the atrium allowing to observe two sections of the endothelium of the same atrium; (**B**) mean endothelium thickness for each group (*n* = 7 to 20; *N* = 2 to 6). Aldosterone treatment produces endothelial thickening in both Trpm4^+/+^ and Trpm4^-/-^ animals. Statistical significance between groups are indicated by *.

The atrial endothelium thickness was measured on LA slices. *Trpm4^+/+^* and *Trpm4^-/-^* animals exhibited similar endothelium thickness with a single cell layer (Figure 5). Aldosterone + salt treatment induced a significant increase of this thickness in both groups (+110% in *Trpm4^+/+^* and +220% *Trpm4^-/-^*) (Figure 5). The endothelial thickness was not significantly different between *Trpm4^+/+^*-AL+S and *Trpm4^-/-^*-AL+S animals.

## 4. Discussion

Our data indicate that aldosterone + salt treatment as well as *Trpm4* disruption induce LA remodeling at both a morphological and electrical level. However, the effect of aldosterone + salt treatment does not seem to add up to that of TRPM4 on several of these parameters since it is still observed in *Trpm4^-/-^* animals. Nevertheless, TRPM4 status influences aldosterone + salt-induced LA AP modifications, arrythmias, and heart rate changes.

Our study used a model of primary hyperaldosteronism [22], which includes a combination of aldosterone administration and enhanced dietary NaCl on uninephrectomized animals. A high salt diet was used because it is known to potentiate aldosterone-induced organ damages [22,23]. According to this, we cannot differentiate the effect of hyperaldosteronemia alone or high salt diet alone. However, basal parameters measured in our study are consistent with other models of hyperaldosteronemia. Aldosteronemia levels measured after one month of infusion was in the range of those observed in patients with hyperaldosteronemia. Indeed, mouse aldosterone plasma level rose from 0.4 × 10^−9^ mol·L^−1^ in control animals (*Trpm4^+/+^* without aldosterone treatment) to 5.9 × 10^−9^ mol·L^−1^ in *Trpm4^+/+^* after one month of minipump infusion while it rose from 0.3 × 10^−9^ mol·L^−1^ in patients with a normal aldosterone level to 1 × 10^−9^ mol·L^−1^ in patients with hyperaldosteronemia and enhanced occurrence of atrial arrhythmias [1]. A previous report indicated a basal aldosterone plasma level of 0.41 × 10^−9^ mol·L^−1^ in *Trpm4^+/+^* mice, in the same range as our results and a similar level of 0.39 × 10^−9^ mol·L^−1^ for *Trpm4^-/-^* mice, indicating no aldosterone impairment due to mouse phenotype [25], as confirmed by our measurements.

At the electrical level, our data indicate a 15% reduction in APD induced by aldosterone + salt treatment in LA form *Trpm4^+/+^* animals, even when it does not reaching significance (*p* = 0.24). Several studies evaluated the effect of prolonged aldosterone treatment on atrial parameters. Hyperaldoteronemia induced during 4 weeks in rat using osmotic pumps produces a 16% shortening of the left atrial AP (similar to those observed in our model) [26]. It also enhances the susceptibility to induced atrial arrhythmias such as atrial fibrillation. It is in line with our observation of an increase of arrhythmias occurrence after an aldosterone infusion of *Trpm4^+/+^* animals. A reduction in atrial APD in *Trpm4^-/-^* mice compare to WT is well documented [12,13,15,16] and is confirmed by our measurements. At the global level, *Trpm4^-/-^* mice exhibit multilevel conduction blocks but also bursts of ectopic atrial activity and a slowing conduction time in atria and His bundle [12]. This might be partly explained by the appearance of arrhythmias such as EAD and DAD that we observed at the cell level in atria from *Trpm4^-/-^* mice.

An interesting finding of our study is that aldosterone produces a lengthening of the atrial AP in the absence of TRPM4 but no significant effect was observed in the presence of the channel. TRPM4 would thus protect against aldosterone-induced AP prolongation. Since Ca^2+^-transient is altered in atria after aldosterone treatment [27], one can postulate that TRPM4 participates through this phenomenon since its activation produces a depolarizing current which influences both the driving force for Ca^2+^ entry and activation of voltage-gated channels. It is also interesting to note that aldosterone treatment increases the occurrence of LA arrhythmias in *Trpm4^+/+^* animals but, on the contrary, reduces their occurrence in *Trpm4^-/-^*. It indicates that TRPM4 contributes to aldosterone-induced arrhythmias even if the mechanism is not yet fully elucidated. Since Ca^2+^ transient is a central element in APD and arrythmias, any modification in the expression of one of its actors could be involved in the differences observed in our experiments, particularly because aldosterone is known to modulate gene expression and because TRPM4 is involved in Ca^2+^ transient regulation. We did not evaluate this point in our experiments but exploring the expression level of TRPM4 under aldosterone + salt stimulation may provide a valuable clue to understand electrical remodeling since it is known that TRPM4 expression influence AP duration and thus arrythmias. In addition, expression or regulation of Ca^2+^-handling proteins such as Na/Ca exchange, ryanodine receptors, Ca^2+^ pump, and voltage gated Ca^2+^ channels may also be influenced by an aldosterone-salt treatment and the presence or absence of TRPM4. Exploring their expression would be a step forward.

Disorganization of Cx43 in atria may also participate in electrical perturbations at the atrial level. Indeed, we observed such disorganization in *Trpm4^-/-^* but not in *Trpm4^+/+^* animals, even in the absence of aldosterone treatment. *Trpm4* disruption is not associated with variation in *Cx43* gene expression at the atrial level, as previously shown at the mRNA level [12]. It indicates that only the organization is altered which is consistent with our observation of Cx43 translation from intercalated disks to the lateral membrane. To our knowledge, this is the first demonstration of a relation between *Trpm4* expression and Cx43 organization. Distribution of Cx43 at the lateral membrane would impair appropriate conduction, leading to arrythmias [28]. We also observed that aldosterone produces a Cx43 disorganization in *Trpm4^+/+^* animals. Aldosterone treatment (24 h) was shown to modulate Cx43 expression in-vitro, on rat ventricular cardiomyocytes in culture, with an effect on conduction velocity within the culture [10]. This was reproduced on several animal models. Cardiac hypertrophy induced by aortic banding in mice produced a decrease in Cx43 expression and induced their disorganization at the junctional plaques [9], which was prevented by treatment with the MR antagonist spironolactone. On the other hand, the infusion of aldosterone in rat, using an osmotic minipump, did not change the expression level of Cx43 in atria [2]. Since in our hands we observed a disorganization at the junctional plaques, it suggests that the effect of aldosterone occurs through a disorganization of Cx43 more than a modulation of protein expression. According to the low level of remaining organized Cx43 at the junctional plaque detected after aldosterone treatment or *Trpm4* disruption, we cannot conclude on a possible cooperation between these two parameters.

Aldosterone treatment had no effect on heart rate in Trpm4^+/+^ animals, similarly to the comparison between patients with or without hyperaldosteronemia [1], but induced a reduction in Trpm4^-/-^ mice. This suggests that TRPM4 might be involved in a compensatory process to counteract a decrease in heart rate induced by aldosterone. In that sense, it was shown that TRPM4 inhibition decreases the heart rate and that its contribution to a heart rate is stronger at a low rate in a model of free-beating isolated mice right atria [29].

At the morphological level, basal parameters of *Trpm4^+/+^* and *Trpm4^-/-^* mice measured in our study are consistent with previous reports on such animals [12,25,30,31,32]. Interestingly, we detected a weak LA dilatation without an increase in the atrial weight in *Trpm4^-/-^* animals compared to *Trpm4^+/+^*. This is, to our knowledge, the first report of LA structural remodeling induced by *Trpm4* disruption. We also observed an increase in cell density in atria from *Trpm4^-/-^* mice, suggesting a similar process to those previously observed at the ventricular level where hypertrophy was attributed to hyperplasia [12,33,34]. Aldosterone-induced atrial dilatation did not appear to be influenced by *Trpm4* disruption in our study even if dilatation was observed by *Trpm4* disruption itself. It indicates that TRPM4 and aldosterone most probably do not participate in the same pathway for this structural remodeling. One can also note that the increase in cell density induced by *Trpm4* disruption was not modified by aldosterone.

The endothelial enlargement observed in our study is challenging. Hyperaldosteronemia induces a variety of endothelial damages which includes genomic and non-genomic pathways leading to inflammation, remodeling, and atherosclerosis [35]. These effects can occur through MR-dependent or independent mechanism but were demonstrated to be MR-dependent in endothelial remodeling [35]. Among these, an effect was reported on ion channels distribution in endothelial cells with an altered expression of the epithelial Na channel [36] or Ca^2+^-activated small conductance K channels [37]. Aldosterone was also shown to promote cardiac endothelial cell proliferation, with an MR-dependent mechanism, after 6 days of infusion in vivo in mice but also ex vivo in human preparations [38]. We did not evaluate the level of TRPM4 expression in our model of hyperaldosteronemia. However, TRPM4 was shown to be expressed in rat and mice endothelial cells [25,39] and its expression is increased in the human umbilical vein cell line (HUVEC) after cell treatment with aldosterone [40]. On the other hand, TRPM4 was shown to be involved in cell proliferation and differentiation in a variety of models. For instance, it prevents the conversion of endothelial cells into fibroblasts [41]. We also recently showed that it promotes human and mouse atrial fibroblast growth in culture [42]. Whether aldosterone-induced increase of atrial endothelial thickness is due to endothelial cell proliferation, hypertrophy or extracellular matrix synthesis remains to be determined. Remarkably, suprarenal abdominal aortic constriction, which produces pressure overload was shown to induce morphological changes (hypertrophy and disarrangement of lines of cells) in the left atrial endothelium of rats associated with an increase of atrial fibrillation susceptibility [43]. Since hyperaldosteronemia is a cause of hypertension [1], this pressure overload may participate in atrial endothelial remodeling in our model.

Note that we did not observed any sign of fibrosis induced by aldosterone + salt treatment in our model. It indicates that, even if cardiac fibrosis was previously reported in hyperaldosteronemia, the electrical and morphological remodeling that we observed in our study is not a consequence of fibrosis but appear earlier in the development of aldosterone-induced cardiac impairment. A longer treatment (more than 28 days) with aldosterone may be needed to induce fibrosis. It should be noted that no sign of cardiac fibrosis was previously reported on *Trpm4^-/-^* mice, similarly to our results [12].

Altogether our data indicate that hyperaldosteronemia and TRPM4 participate in atrial structural and electrical remodeling. It seems that TRPM4 is not directly involved in the LA morphological remodeling induced by hyperaldosteronemia. At the electrical level, the presence of TRPM4 influences the effect of hyperaldosteronemia. The channel could be protective against aldosterone-induced action potential prolongation or heart rate modulation. Furthermore, TRPM4 would promote aldosterone induced atrial arrhythmias. Although the different stages that involved the TRPM4 channel in the effects of aldosterone remain to be determined, our data provide new arguments indicating that TRPM4 is a major target in cardiac arrhythmias.

## Figures and Tables

**Table 1:** Transthoracic echocardiography parameters.

	Trpm4^+/+^-CTRL (*N* = 16)	Trpm4^+/+^-ALDO (*N* = 18)	Trpm4^-/-^-CTRL (*N* = 8)	Trpm4^-/-^-ALDO (*N* = 10)
Left arial diameter (mm)	1.49 ± 0.02	1.95 ± 0.04 * vs. +/+ CTRL	1.63 ± 0.04 * vs. +/+ CTRL	2.02 ± 0.4 * vs. -/- CTRL
Diastolic Inter-ventricular septum (mm)	0.62 ± 0.02	0.66 ± 0.03	0.78 ± 0.05 * vs. +/+ CTRL	0.78 ± 0.04
Diastolic left ventricular posterior wall (mm)	0.72 ± 0.03	0.74 ± 0.3	0.83 ± 0.06	0.82 ± 0.03
Left ventricular telediastolic volume (mL)	0.13 ± 0.01	0.13 ± 0.01	0.15 ± 0.02	0.12 ± 0.01
Systolic septum thickness (mm)	0.83 ± 0.03	0.85 ± 0.04	1.06 ± 0.04 * vs. +/+ CTRL	0.98 ± 0.07
Systolic left ventricular posterior wall thickness (mm)	0.86 ± 0.03	0.91 ± 0.04	1.01 ± 0.06 * vs. +/+ CTRL	0.98 ± 0.06
Left ventricular telesystolic volume (mL)	0.036 ± 0.003	0.041 ± 0.004	0.039 ± 0.002	0.03 ± 0.003 * vs. -/- CTRL
Left ventricular ejection fraction (%)	0.72 ± 0.01	0.70 ± 0.02	0.72 ± 0.03	0.75 ± 0.02
Aorta diameter (mm)	1.52 ± 0.02	1.55 ± 0.02	1.54 ± 0.02	1.55 ± 0.02
Heart rate (bpm)	453 ± 11	429 ± 9	455 ± 16	379 ± 23 * vs. -/- CTRL

Transthoracic echocardiography parameters in 3-month-old mice after 28 days without (CTRL: Control) or with (AL+S) aldosterone treatment. Data are mean ± S.E.M. Statistical significance between groups are indicated by *.

**Table 2:** Morphological mouse parameters.

	Trpm4^+/+^-CTRL	Trpm4^+/+^-ALDO	Trpm4^-/-^-CTRL	Trpm4^-/-^-ALDO
**Body weight (g)**	25.1 ± 0.7 (*N* = 15)	27.1 ± 0.6 (*N* = 13) * vs. +/+ CTRL	25.6 ± 0.6 (*N* = 9)	25.7 ± 0.8 (*N* = 9)
**Heart weight (mg)**	121 ± 6 (*N* = 7)	144 ± 13 (*N* = 5)	131 ± 4 (*N* = 9)	144 ± 1 (*N* = 6) * vs. -/- CTRL
**Tibia length (mm)**	17.9 ± 0.3 (*N* = 8)	18.4 ± 0.2 (*N* = 9)	18.4 ± 0.2 (*N* = 9)	19 ± 0.3 (*N* = 9)
**Heart/tibia (mg/mm)**	6.8 ± 0.3 (*N* = 7)	7.9 ± 0.8 (*N* = 5)	7.1 ± 0.2 (*N* = 9)	7.5 ± 0.2 (*N* = 6)
**Atria weight (mg)**	7.2 ± 0.7 (*N* = 7)	8.4 ± 0.5 (*N* = 5)	6.4 ± 0.4 (*N* = 9)	8.1 ± 1 (*N* = 7)
**Ventricles weight (mg)**	115 ± 5 (*N* = 8)	142 ± 12 (*N* = 6)	125 ± 4 (*N* = 9)	137 ± 1 (*N* = 7) * vs. -/- CTRL
**Right kidney (mg)**	212 ± 17 (*N* = 8)	294 ± 24 (*N* = 9) * vs. +/+ CTRL	209 ± 8 (*N* = 9)	302 ± 16 (*N* = 9) * vs. -/- CTRL
**Lung weight (mg)**	155 ± 9 (*N* = 8)	186 ± 11 (*N* = 8) * vs. +/+ CTRL	158 ± 11 (*N* = 9)	181 ± 11 (*N* = 9)
**Liver weight (mg)**	1020 ± 73 (*N* = 8)	1194 ± 74 (*N* = 9)	939 ± 59 (*N* = 9)	1116 ± 50 (*N* = 9) * vs. -/- CTRL

Organ weight was measured from 3-month-old mice after 28 days without (CTRL) or with (AL+S) aldosterone + salt treatment. Data are mean ± S.E.M. Statistical significance between groups are indicated by *.

## Data Availability

The dataset generated during and/or analyzed during the current study are available from the corresponding author on reasonable request.

## Supplementary Materials

The following are available online at https://www.mdpi.com/2073-4409/10/3/636/s1, Figure S1. Picro Sirius red staining.
