# Supplementary material for: TRPM4 Participates in Aldosterone-Salt-Induced Electrical Atrial Remodeling in Mice
Source: Cells. 2021 Mar 12;10(3):636. doi: 10.3390/cells10030636 (PMC7998432; doi:10.3390/cells10030636)
Supplement: Supplementary file 1 [file cells-10-00636-s001.pdf]

Supplementary Materials

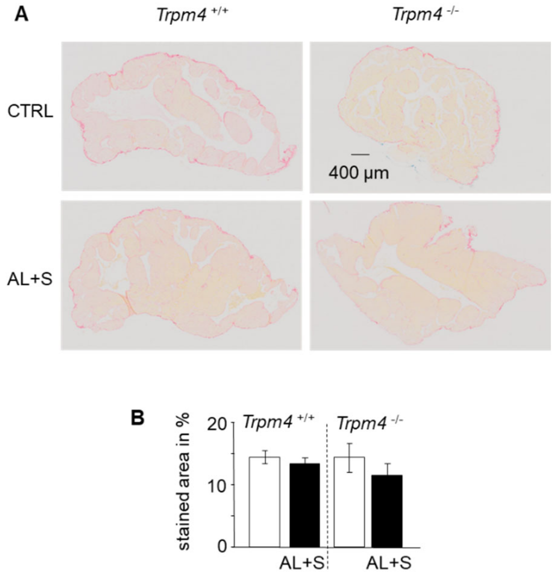

Figure S1. Picro Sirius red staining. **A.** Representative sections (6 µm thickness) of left atrium showing the picro Sirius red staining from *Trpm4*$^{+/+}$ and *Trpm4*$^{-/-}$ animals after 28 days of treatment with or without aldosterone + salt. **B.** Mean relative proportion of stained tissue for each group (*Trpm4*$^{+/+}$-CTRL, n = 24, N = 6; *Trpm4*$^{+/+}$-AL+S, n = 16, N = 4; *Trpm4*$^{-/-}$-CTRL, n = 8, N = 2; *Trpm4*$^{-/-}$-CTRL, n = 12, N = 3). Neither Aldosterone + salt treatment nor *Trpm4* disruption induced fibrosis.
